# Differentiation of Memory CD8 T Cells Unravel Gene Expression Pattern Common to Effector and Memory Precursors

**DOI:** 10.3389/fimmu.2022.840203

**Published:** 2022-05-23

**Authors:** Vanessa Neitzke-Montinelli, Carolina Calôba, Guilherme Melo, Bianca B. Frade, Enzo Caramez, Luciano Mazzoccoli, André N. A. Gonçalves, Helder I. Nakaya, Renata M. Pereira, Miriam B. F. Werneck, João P. B. Viola

**Affiliations:** ^1^Program of Immunology and Tumor Biology, Brazilian National Cancer Institute, Instituto Nacional de Câncer (INCA), Rio de Janeiro, Brazil; ^2^Institute of Biophysics Carlos Chagas Filho, Federal University of Rio de Janeiro, Universidade Federal do Rio de Janeiro (UFRJ), Rio de Janeiro, Brazil; ^3^Institute of Microbiology and Immunology, Federal University of Rio de Janeiro (UFRJ), Rio de Janeiro, Brazil; ^4^Department of Clinical and Toxicological Analyses, School of Pharmaceutical Sciences, University of São Paulo (USP), São Paulo, Brazil; ^5^Hospital Israelita Albert Einstein, São Paulo, Brazil

**Keywords:** CD8 T cell differentiation, immunological memory, cytotoxicity, adoptive cell transfer, immunotherapy

## Abstract

Long-term immunological protection relies on the differentiation and maintenance of memory lymphocytes. Since the knowledge of memory generation has been centered on *in vivo* models of infection, there are obstacles to deep molecular analysis of differentiating subsets. Here we defined a novel *in vitro* CD8 T cell activation and culture regimen using low TCR engagement and cytokines to generate differentiated cells consistent with central memory-like cells, as shown by surface phenotype, gene expression profile and lack of cytotoxic function after challenge. Our results showed an effector signature expressed by *in vitro* memory precursors and their plasticity under specific conditions. Moreover, memory CD8 T cells conferred long-term protection against bacterial infection and slowed *in vivo* tumor growth more efficiently than effector cells. This model may allow further understanding of CD8 T cell memory molecular differentiation subsets and be suited for generating cells to be used for immunotherapy.

## Introduction

Upon activation, naïve CD8^+^ T lymphocytes expand and differentiate into cells with distinct surface phenotypes and survival skills (1, 2). This heterogeneity contributes to a successful immune response, since it leads to the development of an effector component capable of eliminating the trigger antigen, followed by the establishment of a pool of memory T cells that will contribute to a quicker, more efficient response in the event of antigen re-exposure.

Although effector cells are generally short lived and will be mostly eliminated once the antigen is cleared (~90-95%), the remaining population (~5-10%) survives for months to years, generating a population of memory cells that confers the host long-term immunity (3). Different subtypes of memory cells have been identified based on distinct surface markers, function and anatomical locations. The effector memory cells (T_EM_) and tissue resident memory cells (T_RM_) have a nonlymphoid localization, the latter being noncirculating cells, located at potential reinfection sites, such as skin and intestinal, genital and respiratory mucosa (4). These cells contribute to an immediate response due to efficient effector mechanisms, despite the low proliferative capacity and IL-2 production (2, 4). Unlike T_EM_ and T_RM_, central memory T cells (T_CM_) are found at a more undifferentiated state, are located mainly at lymphoid sites and have a greater proliferative potential with low cytotoxic function soon after rechallenge.

The process of T lymphocyte subtype formation, especially memory cells, is still under discussion. In addition to the well-known signals that guide the cellular differentiation program associated with memory, such as IL-7 and IL-15 (5, 6), and with effector cells, such as IL-12 (7, 8), some models were proposed in an attempt to explain the differentiation process and which signaling molecules could be involved in lineage fate decision. The signal-strength model suggests that signal gradient ranging from weak to strong can direct the lymphocyte program fate, resulting in a less (memory) or more differentiated (terminal effector) cell. This model encompasses variation in TCR avidity and growth cytokine signaling strength. Weak TCR activation provided by APC engagement is associated with memory formation (9–11), whereas activation with higher but similar TCR strengths may lead to effector or memory differentiation if it is in the presence of high or low doses of IL-2, respectively (12). The avidity is also likely to impact the duration of the interaction between the T cell and APC. Lengthier signaling is associated with effector cell differentiation, whereas briefer encounters generate memory-like cells (10, 11). Other than signal-strength, the heterogeneity of precursors may dictate the acquisition of effector or memory characteristics, an event that can take place by asymmetric division during which antigen presentation by the APC directs the rearrangement of organelles and cytoplasmic factors such that two distinct cells are generated in the first division: effector and memory from the proximal to the distal portion of the immune synapse, respectively (13, 14). Alternatively, differences in the pool of naïve precursors and their developmental origin may predispose the activated cells to become one cell type or the other (15). The proposed models are probably not mutually exclusive at the population level and take place guided by the combination of particular microenvironmental characteristics, independently of clonal specificity, as seen by the plasticity of expression of the effector-associated Klrg1 receptor (16). Taken together, these findings contribute to the complexity of differentiated T cell populations (17).

Considering the open questions about the mechanisms involved in lymphocyte differentiation, especially regarding the formation of immunological memory, we aimed to understand the processes that orchestrate the differentiation of CD8 T lymphocytes. Moreover, we investigated the level of commitment and therefore plasticity of memory and effector CD8 T cell precursors.

All the presented observations indicate that the mechanisms that orchestrate the differentiation of CD8 T lymphocytes, as well as the level of commitment and therefore plasticity of memory and effector CD8 T cell precursors, are far from being completely understood. Currently, access to memory cell precursors is limited, since *in vitro* memory CD8 T cell differentiation protocols described in the literature have not achieved classical central-memory characteristics, and isolation of memory precursors from *in vivo* infection models rely on the use of *ex-vivo* labeled naïve cells exhibiting transgenic TCRs or poorly defined lineage surface markers, transferred *in vivo* for activation (12, 18). In the present study, we developed an *in vitro* differentiation protocol of polyclonal CD8 T lymphocytes that is certain to contribute to a deeper understanding of the early steps in T cell activation. We generate cells that are molecularly, phenotypically and functionally similar to *in vivo* generated central memory cells. These cells are long-lived *in vitro* and promote long-term immunological protection *in vivo*. Moreover, they are potential tools in immunotherapy protocols since they slow down the growth of established experimental tumors. Evaluation of memory cell precursors differentiated using this protocol allowed us to identify characteristics and genes previously associated with commitment to effector cells. These data suggest that among the currently known “effector signature” are genes common to both effector and memory T cell precursors, allowing us to better define lineage-specific expression patterns. Notably, even though the first moments of T cell activation direct the fate of mature T cells, there is still room for plasticity, and a change in the activation microenvironment is able to divert precursors towards different phenotypes.

## Materials and methods

### Animals

C57BL/6 mice were bred and housed in the animal facility of National Cancer Institute of Brazil (INCA) and OT-I mice, kindly provided by Dr. Karina Bortoluci, were housed in the animal facility of Institute of Biomedical Science, University of São Paulo, Brazil (ICB/USP). Male or female 7 to 11-week-old mice were used in all experiments. Animal experiments were performed in accordance with the Brazilian Government’s ethical and animal experimental regulations. The experiments were approved and conducted according to the animal welfare guidelines of the Ethics Committee of Animal Experimentation from INCA (CEUA process nos. 004/13 and 008/13).

### Cell Culture

Primary murine lymphocytes were cultured in DMEM supplemented with 10% FCS, 1x L-glutamine, 1x streptomycin/penicillin, 1x essential and nonessential amino acids, 1x MEM vitamins, 10 nM HEPES, and 55 μM 2-mercaptoethanol (all from Gibco). P815 was cultured in RPMI supplemented with 10% FCS, 1x L-glutamine, 1x streptomycin/penicillin, 1x sodium pyruvate, and 55 μM 2-mercaptoethanol (all from Gibco). All cell cultures were maintained in a humidified environment containing 5% CO_2_ at 37°C.

### Lymphocyte Purification, Activation and Differentiation

Total CD8 T cells were negatively enriched using Dynabeads Untouched™ mouse CD8 cells kit (Invitrogen™) from inguinal, brachial, axillary and cervical lymph nodes. The purity was greater than 94% in all experiments, as measured by flow cytometry (FACScalibur™, Becton Dickinson, Mountain View, CA, USA) of cells labeled with anti-B220 FITC, anti-CD4 PE (both from BD Pharmingen™), anti-CD8 PerCP.Cy5.5 and anti-CD3 APC antibodies (both from eBioscience™). For naïve CD8 T cells isolation, these cells were negatively selected using the CELLection Biotin Binder kit (Invitrogen™) and anti-CD44 biotin Ab (eBioscience™). For *in vitro* differentiation, naïve or total CD8 T cells or OT-I CD8 T cells (1 x 10^6^ cells/ml) were activated *in vitro* for 48 h with 50 ng/ml (memory) and 1 μg/ml (effector) of plate-bound anti-CD3 plus 1 μg/ml of anti-CD28 (both from BD Pharmingen™). To generate effector cells, IL-12 (10 ng/ml; Peprotech^®^) was administered in one dose at the moment of activation while murine recombinant IL-2 (200 U/ml; Peprotech^®^) was added daily from the second day of culture forward. The analysis was carried out at day 5 or otherwise indicated, doubling the culture medium every day starting on day 3 of culture. To the memory differentiation culture was added 10 ng/ml of IL-7 and IL-15 (Peprotech^®^) for the first three days, followed by IL-15 through the 10^th^ day or indicated time. Additionally, rIL-2 (20 U/ml) was added from day two until the end of memory differentiation. Cultures were doubled from days 3 through 6. From day 8 forward, half of the culture medium was changed every other day without disturbing the cells until the endpoint of the experiment was reached. Alternatively, the *in vitro* memory-like cells were also generated following the protocol described previously (12). Briefly, naive CD8 T cells were cultured with anti-CD3 plus anti-CD28 in presence of rIL-2 (20 U/ml) until the end of memory differentiation (12).

### Flow Cytometry, Sorting and Intracellular Staining

To analyze cell surface proteins levels, 5 × 10^5^ lymphocytes were labeled with the following mAntibodies: anti-B220 FITC, anti-CD4 PE, anti-IFNγ PE (BD Pharmingen™), anti-CD122 PE, anti-CD127 PE, anti-CD25 APC, anti-CD25.AF488, anti-CD3 APC, anti-CD44 FITC, anti-CD62L PE, anti-CD62L PerCP.Cy5.5, anti-CD8 PerCP.Cy5.5, anti-Granzyme B FITC, anti-IgG2a K FITC, anti-IgG2a K PE, anti-Klrg1 FITC (all eBioscience™). For intracellular staining, 1 × 10^6^ cells/ml were stimulated *in vitro* for 6 h with 10 nM *of Phorbol* 12-myristate 13-acetate (PMA) plus 1 μM of ionomycin (both from Calbiochem^®^). Brefeldin A (1:1000; BD Pharmingen™) was added to the culture for the last 2 h. Cells were harvested and stained with anti-CD44 APC and anti-CD62L PerCP.Cy5.5 antibodies. Then, the cells were fixed, permeabilized, and stained with anti-Granzyme B FITC, anti-IFN-γ PE or anti-IL-2 PE antibodies and analyzed by flow cytometry on a FACSCalibur. For the memory subtype analysis, day 10 memory cells generated *in vitro* were sorted using a MoFlo Cell Sorter (Beckman Coulter, Inc) based on the CD62L level. All the flow cytometry data were analyzed using FlowJo^®^ software.

### Animal Infection, Tumor Model and Adoptive Cell Transfer

For LCMV infection, C57BL/6 mice were infected with 2 x 10^5^ pfu of LCMV Armstrong *via* i.p. inoculation. Splenic effector and memory CD8 T cells were analyzed 8 and 33 days later, respectively, through gp33 Tetramer-KAVYNFATC.AF647 (from NIH) anti-CD44 and anti-CD8 Ab staining. For long-term protection experiment, 1 x 10^4^ naïve, *in vitro* effector or memory-differentiated OT-I CD8 T cells were adoptively transferred i.v. into WT C57BL/6 24 h after irradiation (4 Gray). Twenty days after cell transfer, the recipients were infected i.p. with a lethal dose (1 x 10^7^ cfu) of *Listeria monocytogenes* that produces ovalbumin (Lm-ova). Survival of recipient mice was evaluated daily, and the bacterial burden in the spleen was quantified by calculation of the colony-forming units (cfu) on BHI plates when mice became moribund. For anti-cancer immunotherapy, C57Bl/6 mice were inoculated subcutaneously with 0.3 x 10^6^ B16-OVA cells in the right flank. Four days later, mice received 1.5 x 10^6^ effector or memory OT-I CD8 T cells that had been differentiated as described above, intravenously through the tail vein. Two diameters of the tumors were measured and tumor volume (Tv) estimated according to Tv = 0.52 x (width^2^ x length). Animals were euthanized when tumors ulcerated or reached 1000 mm^3^. The experimental endpoint was reached when all animals from a single group were euthanized.

### RNA Extraction and Gene Expression Analysis

Total RNA was extracted using TRIzol LS Reagent (Invitrogen™). After DNase I (Invitrogen™) treatment, the cDNA was synthesized using Superscript^®^ II reverse transcriptase kit with random primers (Invitrogen™). Real-time polymerase chain reactions were performed using TaqMan^®^ probes for *Prdm1* (Mm00476128_m1), *Tbx21* (Mm01351985_m1), *Bcl6* (Mm00477633_m1), *Eomes* (Mm00450960_m1), *Hk2* (Mm00443385_m1), *Tcf7* (Mm00493445_m14331182), *Ezh2* (Mm00468464_m14331182), *Zeb2* (Mm00497196_m14331182) and *Id3* (Mm00492575_m1). *Hprt* (Mm01545399_m1) was used as an endogenous control. All procedures were performed according to the manufacturers’ instructions.

### RNA-seq

Total RNA from effector and memory CD8 T cells differentiated *in vitro* was purified using the RNeasy Plus Mini kit (Qiagen). The RNA yields were quantified using the Qubit RNA HS Assay kit (Invitrogen), and the RNA quality was evaluated with the Agilent 2100 Bioanalyzer (Agilent). The transcriptome libraries were constructed from purified RNA with the Illumina TruSeq RNA Sample Preparation Kit v2 (Set A), AMPure XP Beads (Beckman Coulter Genomics) and SuperScript II Reverse Transcriptase (Invitrogen) according to the manufacturer’s instruction. Validation and quantification of the libraries were performed using the DNA 1000 Agilent Kit (Agilent) and Qubit dsDNA BR Assay (Invitrogen), respectively, according to the manufacturer’s instructions. The transcriptome pools were loaded onto the cBot DNA Cluster Generation System and clustered using the HiSeq Rapid PE cluster v2 Kit (Illumina). The libraries were sequenced as 100-bp paired-end runs on an Illumina HiSeq1500.

Reads were filtered for adapter sequences and trimmed for sequence quality with Trimmomatic version 0.38, mapped to the GRCm28 mouse genome with bowtie2 version 2.3.4.3 and counted with featureCounts from Rsubread R package version 1.32.4. Statistical analysis of RNA-Seq data was performed in R. Differential expression was calculated with DESeq2 (19). Data were visualized using ComplexHeatmap (20) and ggplot2. Gene Set Enrichment Analysis was performed with GSEA (21) using the memory CD8 T cell signature generated from published data (22). Clusters delimited by hierarchical clustering displayed in data visualization were tested with pvclust.

### *In Vitro* Cytotoxicity Assay

To evaluate the cytotoxic ability of *in vitro* differentiated lymphocytes, 5 x 10^6^ cells/ml of target cells (P815) were stained with 10 μM Calcein-AM (Molecular Probes^®^) in RPMI medium for 30 min at 37°C. The assay was performed in V bottom 96-well microtiter plates with target-vs-effector (T:E) ratios ranging from 2:1 to 1:5 maintaining the number of target cells constant (5 x 10^4^ cells/well) in the presence of 1 μg/ml of soluble anti-CD3. The background was evaluated through spontaneous (target in medium alone) and maximum release (target cells lysed in medium plus 2% Triton™ X-100; Sigma Aldrich^®^) as described previously (23).

### Statistical Analysis

For single comparisons, the unpaired Student’s *t*-test was applied, whereas for multiple comparisons, one-way ANOVA with Tukey’s multiple test correction was used. The Mann-Whitney test was used for bacterial burden median comparison. The ordinary two-way ANOVA with Tukey´s multiple comparison test was used for tumor growth analysis. Survival curve was statistically assessed by Log-rank (Mantel-Cox) test. Flow cytometry and mean fluorescence intensity (MFI) analysis were performed using FlowJo 10.1r5 software and analyzed for statistical significance with the unpaired two-tailed Student’s *t-*test using Prism (GraphPad Software). Differences with *p* < 0.05 were considered statistically significant.

## Results

### Low TCR Avidity and a Low Dose of IL-2 Can Induce Long-Term Polyclonal Memory CD8 T Cells *in Vitro*

**Figure 1:**
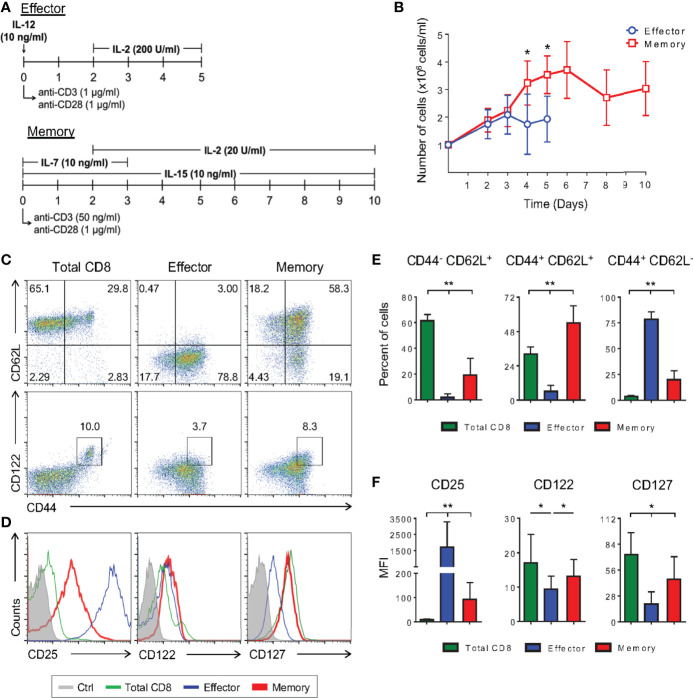
Low TCR Avidity and a Low Dose of IL-2 Can Induce Long-Term Polyclonal Memory CD8 T Cells *In Vitro*. **(A)** Schematic representation of the differentiation regimen for the *in vitro* effector (top) and memory (bottom) CD8 T lymphocytes. **(B)** Number of effector and memory cells during differentiation culture quantified by trypan blue exclusion. **(C, D)** Negatively-selected purified (total) CD8 T cells and *in vitro* differentiated effector and memory CD8 T lymphocytes were labeled with anti-CD44, anti-CD62L, anti-CD122, anti-CD127 and anti-CD25 fluorescently labeled monoclonal antibodies on the 5^th^ and 10^th^ days of culture, respectively. **(E)** Percentage of CD44^-^CD62L^+^, CD44^+^CD62L^+^ and CD44^+^CD62L^-^ CD8 T cells. **(F)** Mean fluorescence intensity (MFI) of anti-CD25, anti-CD122 and anti-CD127 fluorescently labeled cells. All data are shown as the mean ± SD. The (*) indicates *p* < 0.05 and (**) *p* < 0.01 comparing all groups to each other. All results are representative of at least 13 independent experiments. Ctrl: unlabeled (gray); total CD8 (green); effector (blue); memory (red).

Detailed molecular and phenotypic studies of memory CD8 T cell precursors have been precluded by the lack of reliable sources of these cells. *In vitro* cultures have been attempted using low doses of IL-2, which gives rise to memory-like cells that retain some effector functions (12). Alternatively, TCR transgenic cells have been sorted after *in vivo* activation, often elicited by live infection (13). In both cases, the results are biased towards a more effector-prone context or homogeneous TCR specificity. Based on observations independently reported (10, 12, 18, 24), we have established an *in vitro* protocol for memory T cell differentiation where polyclonal CD8 T cells were activated *in vitro* with mild TCR crosslinking through incubation with a low dose of anti-CD3 (50 ng/ml) plus anti-CD28 (1 μg/ml) in the presence of the cytokines IL-2 (20 U/ml), IL-7 and IL-15 (both at 10 ng/ml) (**Figure 1A**). The effector T cell differentiating protocol was carried out with strong TCR engagement (1 μg/ml of anti-CD3 plus 1 μg/ml anti-CD28), IL-12 (10 ng/ml) and high doses of IL-2 (200 U/ml) (**Figure 1A**). Evaluating the kinetics of *in vitro* CD8 T cell activation, cells differentiated in the memory-inducing protocol displayed greater cellular expansion compared to *in vitro* effector cells (**Figure 1B**).

Consistent with what has been previously shown in *in vivo* infection models (25), the *in vitro* effector protocol generated cells that were CD44^+^CD62L^-^, while our optimized, low-avidity memory-inducing protocol mostly generated cells with the lymph node-resident CD44^+^CD62L^+^ phenotype (**Figures 1C, E**, and **Supplementary Figure 1**) (11). Among memory lymphocyte subsets, CD44^+^CD62L^+^ cells represent the central memory type which displays some stem-cell properties such as self-renewal and the ability to give rise to different subsets of cells upon stimulation. These characteristics are given, in part, by the cytokines they are able to respond to, with IL-2 and IL-7 being two important examples. It has been previously shown that CD8 T cells cultured in the presence of only IL-2, at low doses (10 U/ml), differentiate into memory cells (12). By adding IL-7 and IL-15 to the culture regimen we generated cells that were CD25^lo^CD127^hi^CD122^hi^ (**Figures 1D, F**), and able to produce IL-2 and IFN-γ with no production of granzyme B (**Supplementary Figure 2D**). These observations demonstrate these cells to be more similar to *bona fide*, *in vivo*-generated memory cells than previously reported (**Supplementary Figure 2**) (12, 26).

CD8 T lymphocytes from naïve mice present a subpopulation of antigen-inexperienced memory CD8 T cells, CD44^+^CD122^hi^, called homeostatic memory T cells (27). This population, however, does not interfere with the differentiated cellular phenotype in terms of surface markers and production of cytokines in our model, since no differences were observed when sorted CD8^+^CD44^-^ naïve cells were used to start the cultures (**Figure 1C** and **Supplementary Figure 2**).

### Gene Expression Profile of Effector and Memory CD8 T Cells Generated *in Vitro*

Differentiation from naïve T cells to effector and memory lymphocytes is orchestrated by a set of chromatin remodelers, transcription factors and regulators that modulate diverse aspects of this process through changes in the pattern of gene expression (1, 2, 13). Changes in the expression of key transcription factors have been associated with effector and memory phenotypes, and they may therefore correlate with the establishment of each of these phenotypes (1, 2).

**Figure 2:**
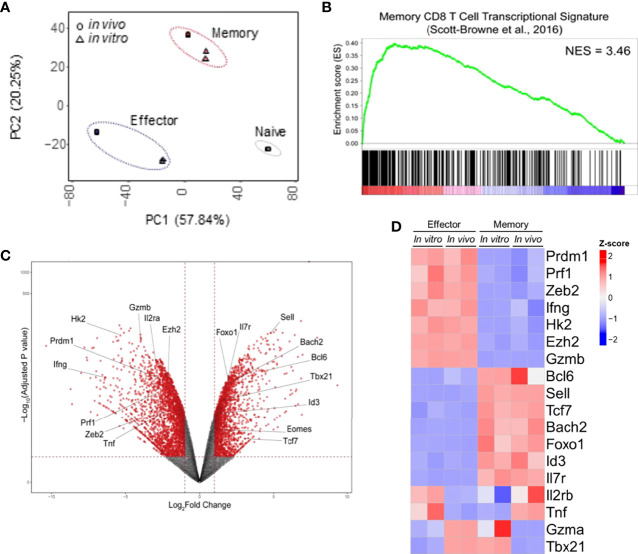
*In Vitro* and *In Vivo*-Generated Memory and Effector CD8 T Cells Are Molecularly Similar. **(A)** Principal component analysis of the RNA-seq data set generated from two independent samples of memory and effector CD8 T cells obtained *in vitro* was performed following the protocol displayed in **Figure 1** and those publicly available from cells generated upon viral infection *in vivo* (LCMV infection, GEO: GSE88987). **(B)** Gene set analysis of gene expression data described in A compared with memory CD8 T cells (22). **(C)** Volcano plot displaying differentially expressed genes between effector and memory CD8 T cells. **(D)** Heat map of differential expression between cells generated *in vitro* and available from *in vivo* model of genes commonly associated with effector or memory CD8 T cell signatures.

**Figure 3:**
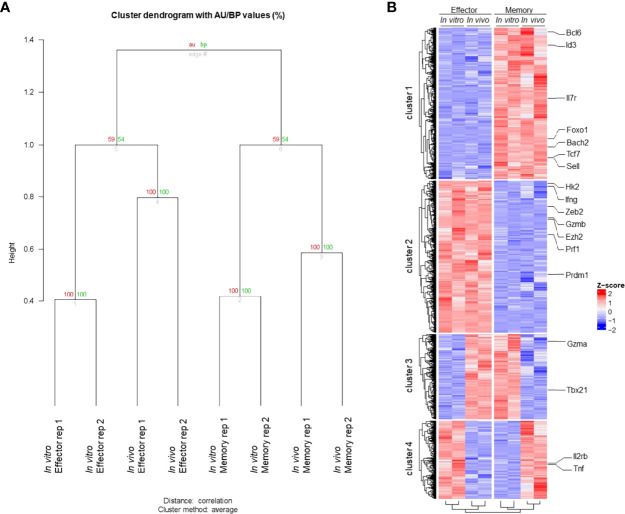
Transcriptome Profile of Memory and Effector CD8 T Cells. **(A)** Clustering global transcriptome of memory and effector CD8 T cells from *in vivo* and *in vitro* samples. The samples were clustered based on the global transcriptome using “average” for cluster method and correlation-based dissimilarity matrix method for distance metric. Two bootstrapping algorithms, AU (approximately unbiased – in red) and BP (bootstrap probability – in green), were used to calculate the similarity between samples and are represented as values (0 to 100) for each branch. **(B)** Hierarchical clustering of the heatmap displaying gene expression from memory and effector CD8 T cells from *in vivo* and *in vitro* samples. Hierarchical column clustering showing samples grouped by cell type (effector and memory) and sample type (*in vitro* and *in vivo*). The hierarchical row clustering showed four bright patterns of gene expression clusters. The criteria to select those genes were a *p* < 0.05 (False Discovery Rate (FDR) < 0.1), and the gene expression is displayed by row as a z-score. All featured labeled genes showed FDR < 0.05.

To access the similarities between memory and effector T cell populations generated *in vitro* using our protocol and *in vivo* following viral infection, we extracted RNA from fully differentiated effector and memory CD8 T cells, performed RNA-seq and compared the results to data previously obtained for LCMV-responsive effector and memory CD8 T cells generated *in vivo* (22). Through principal component analysis, we found that effector and memory cells generated using our protocol showed a good degree of similarity to cells generated *in vivo* after viral infection (**Figure 2A**). Indeed, Gene Set Enrichment Analysis (GSEA) of our data in comparison to the pattern of gene expression of memory cells showed that genes expressed by these cells are enriched in *in vitro*-generated memory cells and were more rarely found in *in vitro*-generated effector cells (**Figure 2B**). Clustering analysis demonstrated that most genes are similarly up or downregulated between cells generated *in vitro* and *in vivo* (**Figure 3A**). The discordant gene expression was found to be associated with proteins important for cell migration, cell adhesion and response chemokines, stimuli that are tightly related to complex activation milieu and were not present in our homogeneous CD8 T cell cultures (**Figure 3B**). However, the pattern of expression of transcription factors and, specifically, the expression of genes previously described as key for memory and effector T cell differentiation were consistent between *in vivo* and *in vitro*-generated memory and effector T cells (**Figures 2C, D**).

**Figure 4:**
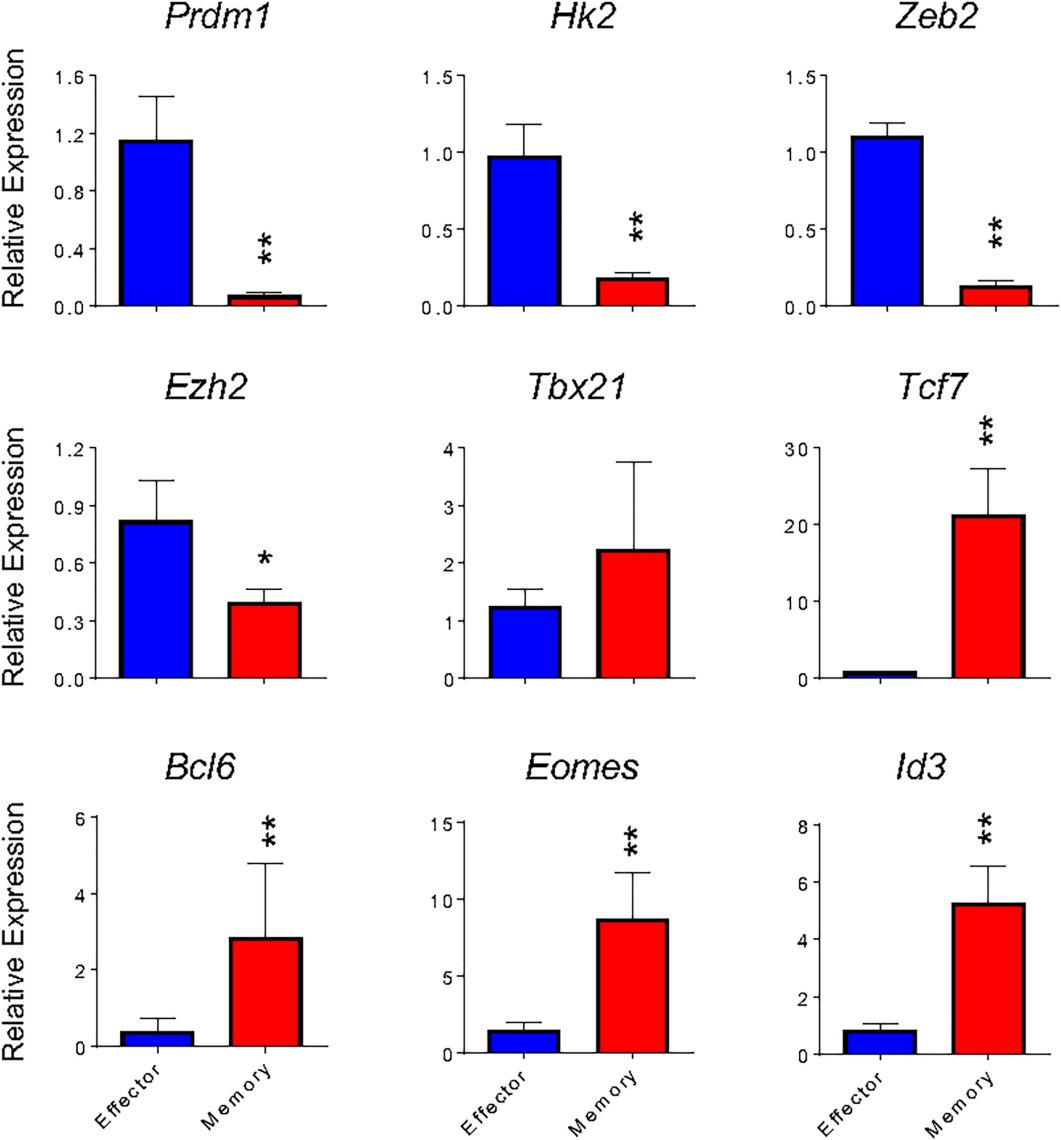
Gene expression of *in vitro* effector and memory CD8 T cells cultures. Total RNA was extracted from effector (blue) and memory (red) T cells differentiated *in vitro* at the 5^th^ and 10^th^ days, respectively, and analyzed by real-time RT-PCR using the TaqMan probe for indicated genes. The data were normalized to the *Hprt* housekeeping gene. Bar graphs represent the mean ( ± SD) of RNA expression analysis. The (*) indicates *p* < 0.05 and (**) *p* < 0.01 compared with the lowest Ct mean obtained from the effector differentiation. All results are representative at least of 4 independent experiments.

As shown in **Figure 2**, *in vitro* and *in vivo*-generated cells showed similar gene expression patterns to respective broadly defined memory and effector T cell signatures. We therefore went on to validate the expression of the most relevant of these genes. The *in vitro* differentiation protocol generates memory cells with low expression of *Prdm1* (encoding Blimp-1 protein) and high expression of *Bcl6*, antagonist regulators of CD8 T cell differentiation that govern the generation of effector and memory T cells, respectively (28, 29). In addition, *Id3* and *Tcf7* (encoding TCF-1 protein) had higher gene expression in memory than effector cells generated *in vitro* (**Figure 4**). These data are in accordance to published results describing that repression of *Id3* by Blimp-1 limits memory formation (30). Moreover, TCF-1 is involved in inducing *Eomes* (Eomesodermin) expression and promoting memory responsiveness to IL-15 (31, 32).

The transcription factors Eomes and T-bet (*Tbx21*) have partially redundant roles in the initial stages of effector generation, leading to an increase in the expression of genes that encode effector proteins (33). However, *Eomes* expression is associated with memory formation (Banerjee et al., 2010). Indeed, it has been shown that the balance between T-bet and Eomes levels is important in defining effectors and memory CD8 T cell fates (34), often making *Tbx21* expression less informative to discriminate effector and memory T cells (13). Corroborating these data, we detected greater expression of *Tbx21* by *in vitro*-differentiated memory cells when compared to their *in vivo*-generated counterparts (**Figures 2C, D**) or *in vitro*-differentiated effector cells (**Figures 2C, D** and **4**), which was accompanied by an even greater differential expression of *Eomes* by *in vitro*-generated memory CD8 T cells (**Figures 2C, D** and **4**). Moreover, the expression of *Zeb2*, a target of T-bet, was upregulated in effector cells (**Figure 4**). The transcription factor Zeb2 cooperates to promote terminal effector program inhibiting *Il7r* and *Il2* expression (35, 36).

Effector cells are already known to perform preferentially aerobic glycolysis, and consequently, some genes associated with this pathway are upregulated, in particular *Hexokinase 2* (*Hk2*) encoding the first rate-limiting enzyme of glycolysis (13, 37). As expected, *Hk2* exhibits 5.7-fold higher expression in effector cells than *in vitro* differentiated memory cells, indicating that the glycolysis pathway is less active in memory cells.

In addition to transcription factors and regulators, effector and memory cells further differ in chromatin state profiles (1, 13, 38). Ezh2, a member of the Polycomb group (PcG) family of chromatin remodeling factors, mediates CD8 T lymphocytes differentiation through epigenetic repression of memory cell-associated genes in terminally differentiated effector cells (13). Consistently, *in vitro*-differentiated memory cells showed lower expression of *Ezh2* (**Figure 4**). Taken together, memory and effector CD8 T cells generated *in vitro* following the protocols herein described exhibited a gene expression profile similar to that defined *in vivo* and previously reported.

### *In Vitro-*Differentiated Memory-Like CD8 T Cells Are Able to Generate Effector-Like Cells Upon Restimulation and Phenotypically Similar to Virus-Induced Memory Cells

**Figure 5:**
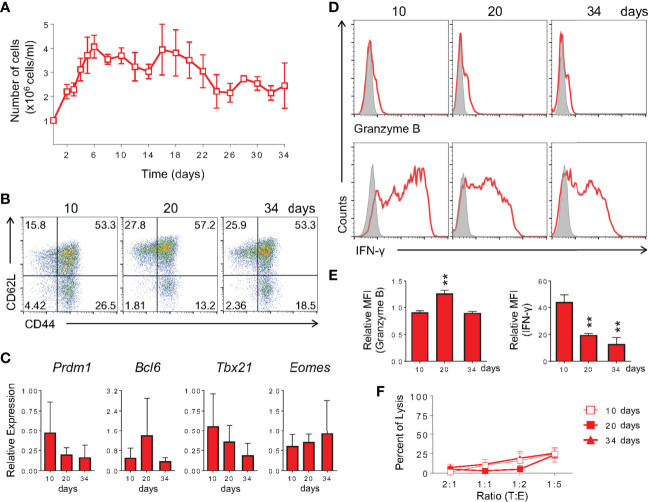
Memory CD8 T Cells Differentiated *In Vitro* Are Long-Lived. **(A)** Memory CD8 T cell cellularity along 34 days of culture. Cells were cultured as described in **Figure 1** until day 10, at which point IL-2 and IL-15 were added every other day until analysis. The number of cells was quantified by trypan blue exclusion. **(B)** Analysis of expression of anti-CD44 and anti-CD62L by *in vitro* memory cells at the 10^th^, 20^th^ and 34^th^ days of culture. **(C)** Real-time RT-PCR assay analysis using TaqMan probe for *Prdm1*, *Bcl6*, *Tbx21 and Eomes* genes. The data were normalized to the *Hprt* housekeeping gene and compared with the lowest memory Ct mean at day 10. **(D, E)** Cells were challenged with PMA and ionomycin for 6 h and ICC stained for IFN-γ and granzyme B detection. The relative MFI was obtained from its respective isotype control MFI normalization. **(F)** Anti-CD3 dependent cytotoxicity assay of *in vitro*-generated memory cells at 10 (square), 20 (filled square) and 34 (triangle) days of culture. All data are shown as the mean ± SD. The (**) indicates *p* < 0.01 compared with day 10^th^. All results are representative of three independent experiments. Ctrl: Mouse IgG2a isotype control (gray); memory (red).

Since the study of memory T cells and their subsets commonly takes place late after initial T cell activation, at a moment when the original antigen has often been cleared and the persistence of reactive cells can be associated with immunity (2, 3), we tested whether the memory cells generated by our protocol were stable and maintained their characteristics after long-term cultures. We therefore extended the *in vitro* memory protocol to 34 days of culture with continued exposure to IL-15 and low IL-2 cytokines. Throughout the culture, cellularity was relatively constant, as well as the maintenance of surface markers and gene expression profile (**Figures 5A–C**). After restimulation with PMA plus ionomycin, long-term (30+ days) *in vitro*-generated memory CD8 T cells produced granzyme B at similar levels to those at day 10 of culture (**Figures 5D, E**). A gradual decrease in IFN-γ levels was observed after 20 and 34 days of differentiation (**Figures 5D, E**). No killing of labeled target cells was detected following this protocol (**Figure 5F**). As for the effector differentiation protocol, cell culture was stable from day 5 through day 7, starting to plunge at day 8 and showing very low viability at day 10 (data not shown). We therefore used day 5 cultures in all experiments.

**Figure 6:**
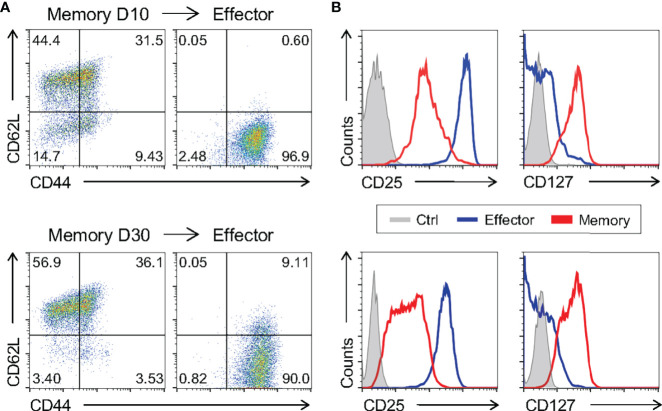
Memory CD8 T Cells Differentiated *In Vitro* Are Capable of Generating Effector Cells. The ten- and thirty-day memory CD8 T cells generated *in vitro* were reactivated applying an adapted model of the *in vitro* effector differentiation protocol for three days of culture. **(A, B)** The memory cells cultured for 10 days (above) and 30 days (below) and the effector cells reactivated from their respective memory were labeled with CD44, CD62L, CD127 and CD25 fluorescently labeled monoclonal antibodies. All results are representative of two or three independent experiments. Ctrl: unlabeled (gray); effector (blue); memory (red).

To further test the similarity of *in vitro-*generated memory cells to bona fide *in vivo*-differentiated ones, we restimulated fully differentiated 10 day- and 30 day-cultured memory cells and evaluated effector vs. memory surface markers. *In vitro* 10 and 30-day memory lymphocytes were able to give rise to effector T cells when changed to and further cultured in effector-biased culture conditions for an additional three days (**Figures 6A, B**). Taken together, these data showed that the phenotype of memory cells generated *in vitro* is stable throughout the culture and that they retain the ability to generate effector-like cells upon restimulation. We analyzed cells after 10 days of culture in further experiments.

**Figure 7:**
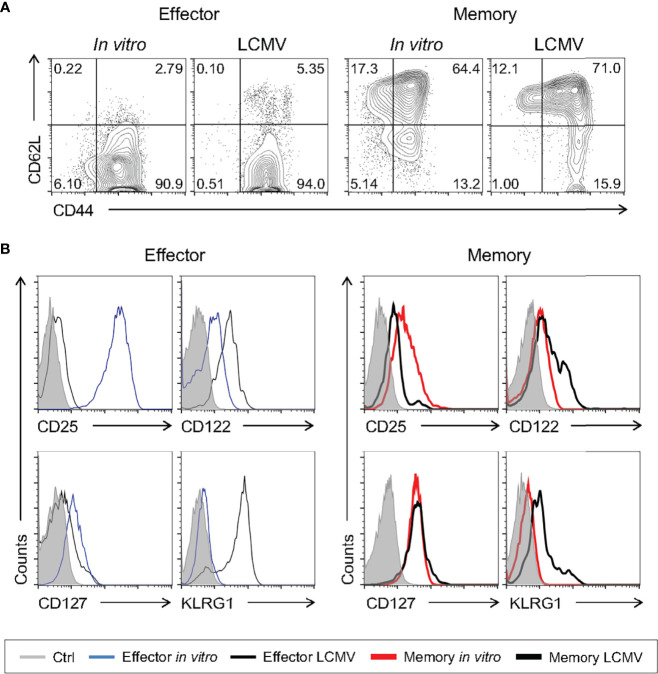
*In Vitro*-Generated Memory-Like CD8 T Cells Are Phenotypically Similar to Virus-Induced Memory Cells. WT mice were infected i.p. with 2 x 10^5^ PFU of LCMV Armstrong strain. The spleen cells were harvested 8 (effector) and 33 (memory) days after infection and compared to the respective *in vitro* CD8 T cell differentiation protocol. **(A)** Comparison between the levels of CD62L and CD44 in gp33-tetramer^+^ effector and memory CD8^+^ T cells from the LCMV infection. Lymphocytes were labeled with anti-CD44 and anti-CD62L fluorescently labeled monoclonal antibody. **(B)** Histograms of anti-CD25, anti-CD122, anti-CD127 and Klrg1 staining from unlabeled (gray), effector (thin black) and memory (thick black) cells from LCMV infection (gp33-tetramer^+^CD8^+^) and effector (thin blue) and memory (thick red) cells from the *in vitro* differentiation protocol. The results are representative of 5 to 11 infected animals.

Even though we have shown that memory cells differentiated *in vitro* are molecularly and phenotypically similar to previously published signatures, we went on to test the pattern of surface markers of cells generated in our study to those generated *in vivo* after viral infection. To generate memory CD8 T cells *in vivo*, WT mice were infected with a sublethal dose of lymphocytic choriomeningitis virus (LCMV) Armstrong strain, a classic model of acute viral immune response. For evaluation of antigen-specific effector and memory CD8 T lymphocytes, gp33-tetramer-positive CD8 T cells were quantified 8 and 33 days after infection, respectively (39, 40). Consistent with previous findings (39), effector cells generated *in vivo* after LCMV infection were CD44^+^CD62L^-^, while *in vivo*-differentiated memory cells displayed two distinct populations according to CD62L expression, effector (CD44^+^CD62L^-^) and central (CD44^+^CD62L^+^) memory (**Figure 7A**). As described above, these phenotypes were similar to those of the present *in vitro*-generated effector and memory cells (**Figures 1** and **7A**). The patterns of CD122 and CD127 were comparable among *in vivo*- and *in vitro*-activated CD8 T cells differentiated using the memory-biased protocol (**Figure 7B**). However, Klrg1^+^ cells were not detected after *in vitro* memory or effector culture (**Figure 7B**), suggesting that the upregulation depends on additional signaling circuits elicited upon *in vivo* viral infection. We did not detect considerable expression of CD25 in cells generated *in vivo* as expected due to its transient expression in the LCMV model (41). Collectively, the memory phenotype generated *in vitro* is comparable to T memory cells obtained from a classical *in vivo* viral-induced immune response.

### *In Vitro*-Induced Memory Precursors Express Some Genes Classically Associated With the Effector Phenotype Before Complete Memory Differentiation

Evaluating the early changes in gene expression profile of CD8 T cells differentiating towards the memory phenotype is challenging due to the difficulty in separating these cells from early effector precursors. Since we have shown that the memory cells generated by our *in vitro* differentiation protocol have a molecular, phenotypic profile similar to memory cells generated *in vivo*, we can now start to address these questions. To achieve this goal, we developed a kinetic profile of differentiating cells, analyzing samples daily throughout the established memory protocol. The surface phenotype and expression pattern of key molecular markers of differentiating cells were evaluated.

**Figure 8:**
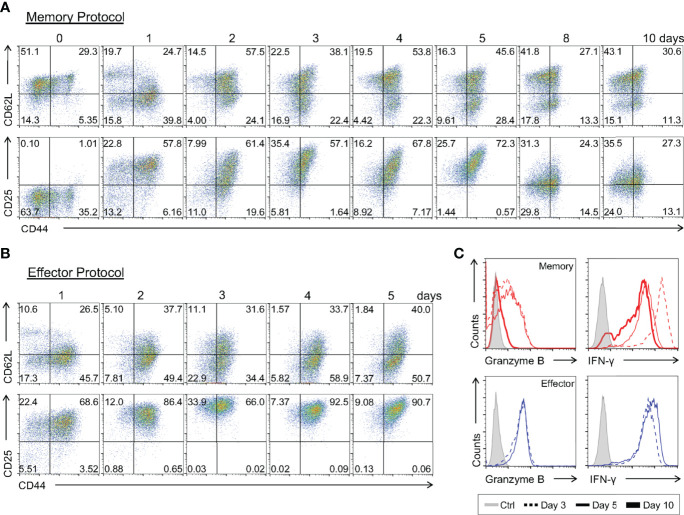
Kinetics of the Immunophenotypic and Functional Profiling of *In Vitro-*Differentiating Memory and Effector CD8 T Cells. Total CD8 T cells and activated lymphocytes using the memory **(A)** or effector **(B)** protocols were stained with anti-CD44, anti-CD62L and anti-CD25 fluorescently labeled antibodies. **(C)** Lymphocytes at day 3 (dotted), 5 (thin) and 10 (thick) of culture were challenged with PMA and ionomycin for 6 h and ICC stained for IFN-γ and granzyme B quantification. All results are representative of 3 independent experiments.

We observed an increase in CD44 and loss of CD62L as early as one day after CD8 T cell activation (**Figure 8A**). However, a CD62L^+^ population was already detected from the 2^nd^ day of culture on until day 10 (**Figure 8A**). The CD127 marker, which was high in naïve CD8 T cells, was downmodulated upon activation and represented around day 8 of culture (**Supplementary Figure 3A**), possibly as a consequence of negative feedback regulation driven by IL-7 signaling (42).

Interestingly, we detected a gradual upregulation of CD25 early during the differentiation regiment, reaching a peak 3 days after T cell activation (**Figure 8A** and **Supplementary Figure 3A**). This upregulation was synchronous with that of effector T cells differentiated *in vitro*, although it had a lower MFI from the 2^nd^ day of culture on (**Figure 8B**). Then, starting on the 8^th^ day of culture, we observed a sharp decrease in CD25 surface presentation, culminating in the characteristic CD25^lo^ phenotype of the memory CD8 T cell population (**Figure 8A** and **Supplementary Figure 3A**). Concomitant to the upregulation of CD25, we saw that memory CD8 T cell precursors were capable of producing high levels of IFN-γ and intracellular Granzyme B upon stimulation, comparable to the findings obtained for effector cells generated *in vitro* (**Figure 8C** and **Supplementary Figures 3B**), and to previously reported work (12). However, when the culture was extended to 10 days a reduction in the production of Granzyme B was observed (**Figure 8C**), similarly to that observed, at the expression level, in memory CD8 T cells analyzed *ex-vivo* (**Figures 2C, D**) (22). These data show that the weak TCR signaling responsible for memory CD8 T cell-biased differentiation is sufficient to elicit expression of effector-associated genes (43), such that memory precursors display some effector-associated characteristics before complete memory differentiation, as proposed by other groups (44, 45).

**Figure 9:**
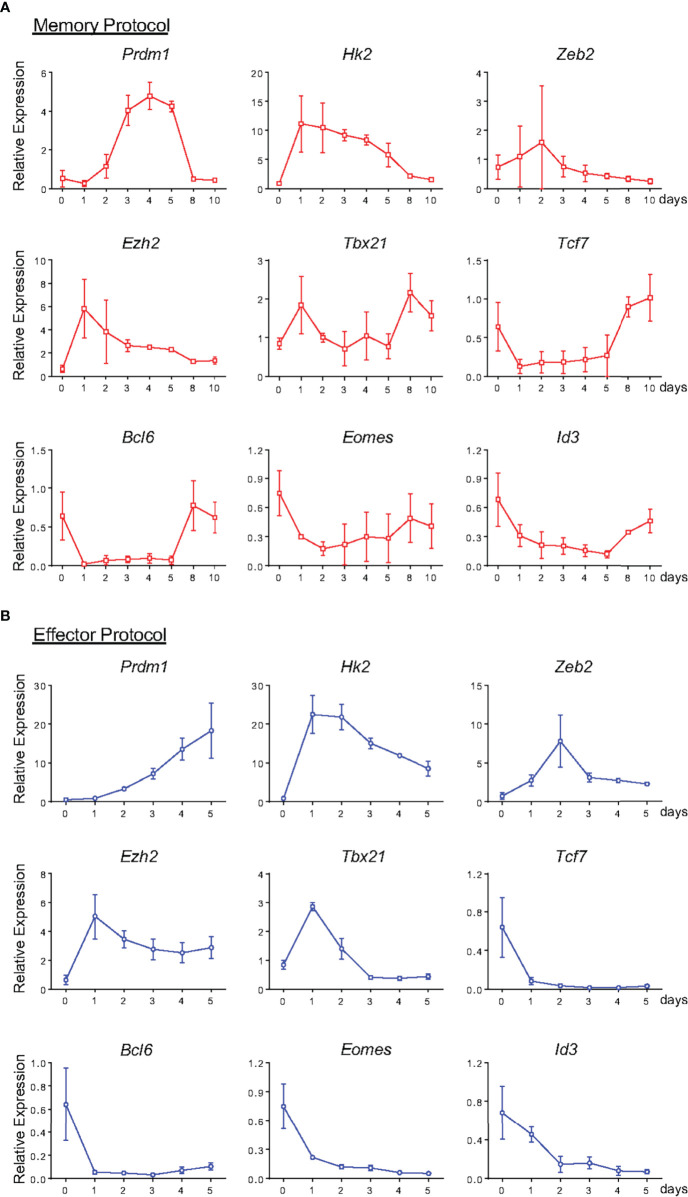
Gene expression profile of Memory and Effector CD8 T cells at Different Stages of *In Vitro* Differentiation. From **Figure 8**, lymphocytes from memory **(A)** and effector **(B)** protocol were analyzed by the real-time RT-PCR assay using TaqMan probe for the indicated genes. The data were normalized to the *Hprt* housekeeping gene, and the reference was the lowest Ct mean obtained from day 0 of differentiation. Bar graphs represent the mean ( ± SD) of RNA expression analysis. All results are representative of 3 independent experiments.

We went on to characterize the pattern of expression of key genes associated with the effector and memory signatures at early stages of memory differentiation (**Figure 9A**). Investigating the hallmarks of the memory profile, we observed a sudden downregulation of *Tcf7*, *Bcl6*, *Eomes* and *Id3* one day after activation. *Eomes* expression remained constant from this time point forward, whereas the other genes showed an upregulation starting on the 8^th^ day of culture (**Figure 9A**). Evaluation of the same gene set in effector precursors revealed a complete downmodulation of these genes throughout differentiation (**Figure 9B**). Surprisingly, when examining the expression regulation of effector-related genes, two groups could be distinguished: one with a very early upregulation consisting of *Prdm1, Hk2 and Ezh2*, and another with expression that remained close to naïve levels and therefore lower than that detected in effector cells, namely, *Zeb2* and *Tbx21* (**Figures 5** and **9A**). As noted in the phenotypic and functional analysis, memory precursor cells expressed genes previously assigned to the effector T cell signature.

### Effector and Memory CD8 T Cell Precursors Are Plastic

It has been demonstrated that differences in the quality of TCR signaling bias CD8 T cell differentiation (11). Indeed, cells prone to weaker signals differentiate towards the memory phenotype, whereas stronger signals yield effector CD8 T cells. Cytokines also direct T cell differentiation, which are sensitive not only to the pool of cytokines present at the moment of activation and throughout differentiation, as shown for IL-7/IL-15 or IL-12, but also to the quantity of cytokines available, as observed for IL-2 (1, 2, 12). This knowledge was incorporated in the protocols herein described. However, we demonstrated that effector and memory CD8 T cell precursors upregulate genes that are common between the two populations (**Figures 9A, B**), genes previously associated with the effector phenotype (1, 2). We therefore asked whether effector and memory CD8 T cell precursors remain plastic after the initial steps of activation, or if, despite the similarities, naïve cells are determined to become one cell type or the other at the moment of activation.

**Figure 10:**
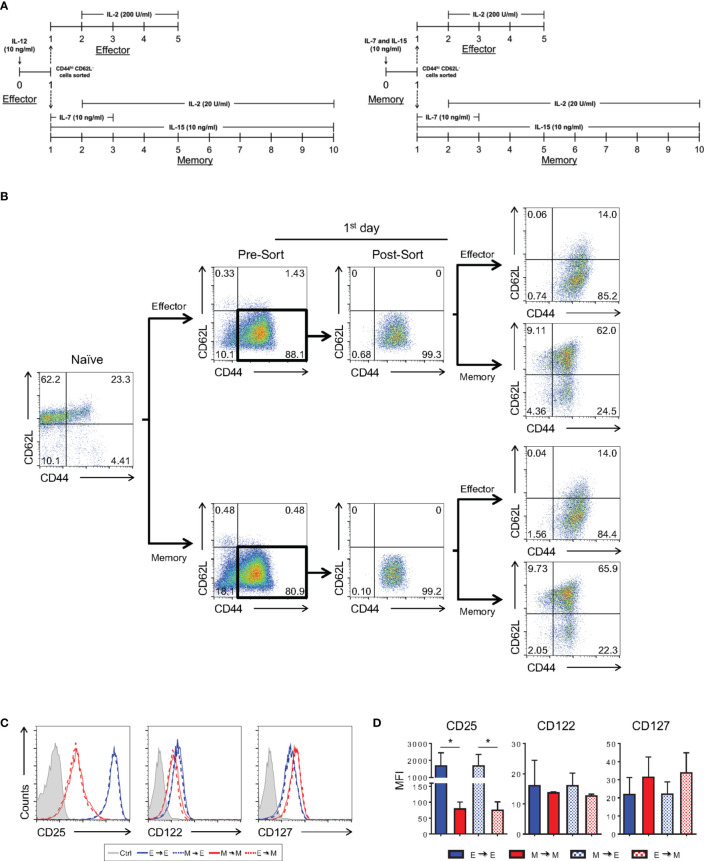
Plasticity of *In Vitro* CD8 T Cell Differentiation in the Early Stages after Activation. **(A)** Schematic representation of the differentiation regimen upon activation and after cell sorting. Timeline on the left depicts cultures that started as effector cells and on the right, that started as memory cells. **(B)** Flow cytometric analysis according to cell-labeling with anti-CD44 and anti-CD62L fluorescently labeled antibodies along the experiment. Briefly, 24h after activation, CD8 T lymphocytes under *in vitro* effector or memory conditions were sorted based on CD44^hi^CD62L^-^ staining. The sorted cells were maintained in the same differentiation condition (control) or subjected to a different protocol (memory or effector), completing the remaining 24 h of the respective activation regiment. The effector and memory cells were analyzed at day 5 and 10 of culture, respectively. **(C)** *In vitro*-differentiated effector and memory CD8 T lymphocytes were labeled with anti-CD25, anti-CD122 and anti-CD127 fluorescently labeled monoclonal antibodies on days 5 and 10 of culture, respectively. **(D)** MFI of anti-CD25, anti-CD122 and anti-CD127 labeling. All data are shown as the mean ± SD. The (*) indicates *p* < 0.05 compared with the effector. All results are representative of three independent experiments. Ctrl: unlabeled (gray); effector to effector (blue); memory to memory (red); memory to effector (blue and white) and effector to memory (red and white).

To achieve this goal, we activated CD8 T cells with a high anti-CD3 concentration in the presence of IL-12 or with a low anti-CD3 concentration in the presence of IL-7 and IL-15, following our effector and memory protocols, respectively (**Figure 10A**). The following day, cells activated using the effector protocol were sorted and transferred such that they remained cultured under the same conditions (high anti-CD3 stimulation and effector cytokine cocktail) or changed to memory-biased conditions (low anti-CD3 stimulation and memory cytokine cocktail) for an additional day, completing the 2-day TCR stimulation (**Figures 10A, B**). The same procedure was performed for cells initially activated in memory-biased conditions. Cultures were allowed to progress for 5 (effector) or 10 (memory) days, after which we assessed their phenotype. As shown in **Figure 10**, independent of the initial stimulus received by naïve CD8 T cells, the phenotype acquired depended on the continuation of the stimulatory conditions. Therefore, the process of CD8 T cell differentiation depended on a sequence of changes that was biased in a plastic manner by the combination of the strength of the TCR signal and the cytokine milieu in which the differentiation process occurred. The cells maintained their susceptibility to changes in this environment, such that the phenotype acquired best suited the immunological needs of the host.

**Figure 11:**
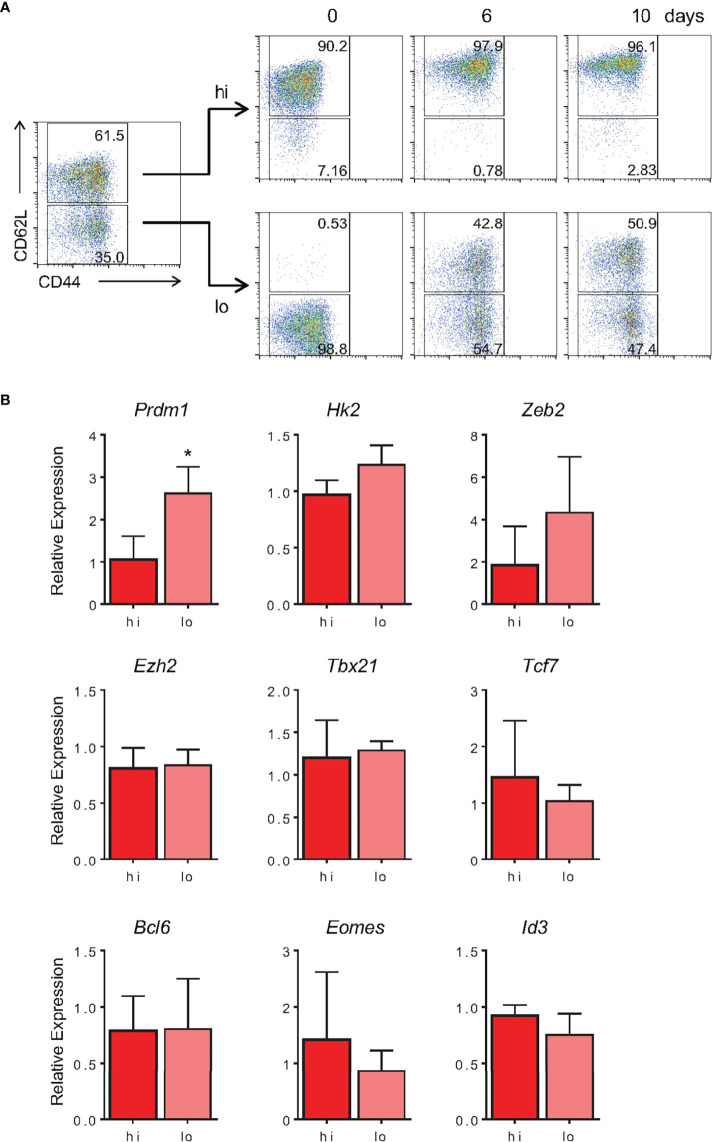
*In Vitro*-Generated T_EM-Like_ Are Able to Give Rise to T_CM-Like_ Cells in Culture. **(A)** Memory CD8 T cells generated *in vitro* were sorted according to CD62L fluorescently labeled monoclonal antibody staining at day 10 of culture. After sorting, CD62L^lo^ and CD62L^hi^ were cultured separately, maintaining the same cell concentration at day 10 of culture. The CD44 and CD62L markers were evaluated on the same day, 6 and 10 days after sorting. The results are representative of two independent experiments. **(B)** Expression of noted genes by sorted CD62L high or low memory cells after 10 days *in vitro* was quantified by real-time RT-PCR using TaqMan probes. Data were normalized to the *Hprt* housekeeping gene, and the reference was the lowest Ct mean obtained from the CD62L^hi^ population. Bar graphs represent the mean ( ± SD) of the RNA expression analysis. The (*) indicates p < 0.05. The results are representative of three independent experiments.

As shown by our group and others, the memory differentiation process, both *in vitro* and *in vivo*, resulted in heterogeneously CD62L-expressing cells (**Figures 1C** and **7A**). Historically, these cells have been referred to as effector (CD62L^lo^) and central (CD62L^hi^) memory CD8 T lymphocytes. The nature of the CD62L^lo^ effector memory (T_EM_) population is still highly discussed, and it is not clear whether population is terminally differentiated or represents a prior step in the formation of central memory cells (T_CM_) (40, 46). In an effort to track the formation of the CD62L^hi^ subpopulations, 10-day memory CD8 T cells generated *in vitro* were sorted based on CD62L expression (CD62L^hi^ and CD62L^lo^) and cultured separately in the presence of IL-2 and IL-15. Starting six days after sorting, a CD62L^hi^ population could be identified in the CD62L^lo^-culture (**Figure 11A**). The reciprocal phenomenon was not observed, suggesting that CD62L^hi^ central memory-like cells, when cultured with cytokines and in the absence of specific TCR engagement, maintain the capacity of self-renewal but are stable and not able to generate CD62L^lo^ CD8 T cell subsets. However, as shown above, strong restimulation of *in vitro*-differentiated memory cells with signals 1, 2 and cytokines promote homogenous activation, downregulation of CD62L expression and acquisition of the effector phenotype (**Figures 8**, **9**).

Evaluating the gene expression profile of these subpopulations, we observed no differences in expression of genes associated with the memory signature, namely, *Tcf7*, *Bcl6*, *Eomes* and *Id3* (**Figure 11B**). Among genes associated with the effector signature, we observed differences in the amount of *Prdm1* expression, which was 2.8-fold higher in T_EM_ compared with T_CM_ (**Figure 11B**). These results indicate that, despite the similarity in expression of several key genes related to the memory vs. effector phenotypes, CD62L^lo^ cells, typically referred to as T_EM_, were able to give rise to CD62L^hi^ (T_CM_) cells in culture. In contrast, T_CM_ were able to self-replicate and maintain their pool in a cytokine-based culture; they represented a stable population and did not give rise to CD62L^lo^ memory or effector CD8 T cell subsets, at least in the absence of further TCR triggering.

### Memory-Like CD8 T Cells Generated *in Vitro* Promote Long-Term Protection Against Lethal *in Vivo* Bacterial Challenge and Reduce the Growth of Established Subcutaneous Experimental Melanoma

We confirmed the efficiency of our *in vitro* memory CD8 T cell protocol upon comparison of their molecular and phenotypic profiles to previously established hallmarks and to *in vivo*-differentiated cells using a classical model of acute viral infection. Nevertheless, we went on to test their functional capacity when compared to effector cells.

**Figure 12:**
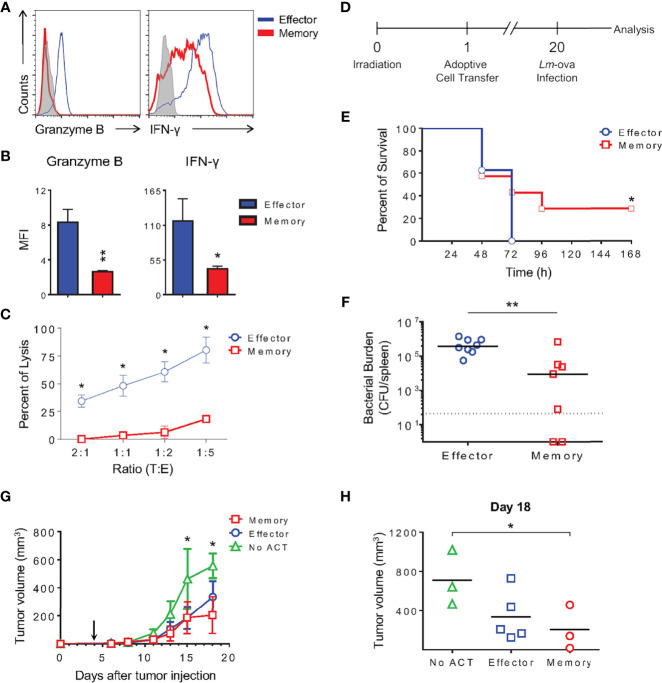
Memory-Like CD8 T Cells Generated *In Vitro* Are Functional, Promote Long-Term Protection Against Lethal *In Vivo* Bacterial Challenge *In Vitro* and Reduce Tumor Progression. **(A)** Effector and memory lymphocytes were stimulated with PMA and ionomycin for 6 h followed by ICC staining for IFN-γ and granzyme *B*. **(B)** Bar graphs represent the MFI mean ( ± SD) of IFN-γ and granzyme B from *in vitro* differentiated lymphocytes. **(C)** Anti-CD3 dependent cytotoxicity assay of effector or memory cells. The results are representative of three independent experiments. **(D)** Schematic representation of long-term protection experiment involving *Listeria monocytogenes* producing ovalbumin (*Lm*-ova) infection. **(E)** Survival curve of mice that received 1x10^4^ effector or memory OT-I CD8 T lymphocytes generated *in vitro* 20 days prior to *in vivo* challenge. The (*) indicates *p* < 0.05 compared with the effector group. The results are representative of two independent experiments. N= 7 to 8 animals per group. **(F)** Median of bacterial burden from the spleen of infected recipients. The (**) indicates *p* < 0.01 compared with the effector group. The results are representative of two independent experiments. N= 7 to 8 animals per group. Effector (blue); memory (red). **(G, H)** C57Bl/6 mice were inoculated with 0.3 x 10^6^ B16-OVA melanoma cells in the right flank 4 days prior to transfer of 1.5 x 10^6^ *in vitro-*differentiated effector or memory OT-I T cells intravenously (arrow). Tumors were measured and the volumes calculated as described in the Methods. The (*) indicates *p* < 0.05 for the difference between tumor size in animals NO ACT and those that received memory OT-I cells. The results are representative of two independent experiments. N = 4 to 5 animals per group. **(G)** Average of tumor volume for each group of mice that did not receive activated OT-I cells (NO ACT), receiving effector or memory OT-I cells. **(H)** Analysis of tumor volume 18 days after tumor inoculation.

Memory and effector cells generated *in vitro* were further stimulated with PMA and ionomycin for 6 h, at which point production of effector molecules were evaluated. Memory cells produced no granzyme B and lower levels of IFN-γ compared with effector CD8 T cells during a short boost (**Figures 12A, B**). Accordingly, only minimal killing was detected upon incubation of memory cells with p815 target cells in an antibody-dependent redirected lysis assay (18.5% of death at 1:5 target:effector T lymphocytes; **Figure 12C**).

The classical function of immunological memory is the promotion of long-term protection in the event of antigen re-exposure. To test whether memory cells differentiated *in vitro* could confer immunity against a lethal challenge to naïve hosts, we used ovalbumin-producing *Listeria monocytogenes* (*Lm*-ova) in an *in vivo* infection model. OT-I TCR transgenic effector and memory CD8 T cells generated *in vitro* were adoptively transferred to irradiated naïve WT recipient mice. Phenotypic analysis of differentiated OT-I cells showed no differences from the polyclonal CD8 T lymphocytes described herein (data not shown). Twenty days later, these mice were challenged with a lethal dose of *Lm*-ova. Survival of the infected mice was monitored and bacterial burden quantified in the spleen of moribund animals (**Figures 12D–F**). Mice that received effector cells 20 days prior succumbed 72 h after infection, whereas 28.6% of the animals receiving OT-I memory cells differentiated *in vitro* were protected (**Figure 12E**). Moreover, the animals receiving memory OT-I CD8 T cells also exhibited a lower total bacterial load in the spleen, indicating that these cells were able to control *in vivo* bacterial growth (**Figure 12F**).

Among the new therapies developed to reduce tumor growth and induce remission, cellular transfers of autologous tumor-responding lymphocytes were quickly being improved. One limiting step resided in generating the large number of reactive cells needed for transfer to tumor-bearing patients. Our protocol of *in vitro* differentiation yields approximately 50 times more memory cells and 16 times more effector cells than the number of naïve cells used for activation (**Figure 1B**). We next tested the capacity of *in vitro*-generated cells to modulate tumor growth in a murine model of melanoma transplant.

We modeled the clinical setting inoculating C57Bl/6 mice with melanoma expressing ovalbumin as a surrogate antigen four days prior to T cell transfer. Tumor was inoculated subcutaneously, and after four days of growth, *in vitro*-differentiated effector or memory CD8 T cells expressing OT-I TCR were transferred intravenously. Tumor size was measured, and the mean volume within the experimental groups was plotted over time (**Figure 12G**). We found that both effector and memory cells protected the animals, reducing the growth rate of the tumor (**Figures 12G, H**). Tumors progressed similarly in all groups until 8 days posttumor injection, which was 4 days after OT-I cell transfer. Starting 11 days after tumor inoculation, we could observe a 60% reduction in tumor growth in mice that received either effector or memory *in vitro*-differentiated cells, achieving statistical significance 15 days after tumor transfer (**Figure 12G**). Interestingly, memory cells seemed more efficient in reducing tumor growth than effector cells, as demonstrated by data collected 18 days posttumor transfer (**Figure 12H**). These observations corroborate the beneficial therapeutic impact of memory CD8 T cells and suggest that the protocol described herein is feasible for immunotherapy, potentially improving current results examining this topic.

## Discussion

The adaptive immune response involves the clonal expansion of antigen-specific effector lymphocytes capable of recognizing and eliminating the immunogenic antigen. Once the immune response is ceased, a population of long-lived lymphocytes called memory cells can still be detected. The question of how naïve CD8 T cells are induced to become effector or memory cells has long been debated, but not clearly defined, mostly due to technical difficulties in isolating memory CD8 T cell precursors *ex vivo* (1, 13, 16, 27). In the present work, we developed a differentiation protocol of central memory cells by *in vitro* activation of polyclonal total CD8 T lymphocytes from naïve mice that resembles *in vivo* generated memory cells according to molecular (**Figures 2**–**4**), phenotypic (**Figures 1**, **7**, and **Supplementary Figure 2**) and functional (**Figure 12** and **Supplementary Figure 2**) criteria, including longevity (**Figure 5**), plasticity (**Figures 10**, **11**) and long-term protection of naïve hosts against lethal bacterial challenge and tumor growth upon transfer (**Figure 12**). With this tool, we were able to profile early memory CD8 T cell precursors and address the characteristics of both maturing (**Figures 8**–**10**) and mature (**Figures 2**, **3** and **11**) memory T cell subsets.

The initial observation that CD4 T cell help is essential for CD8 T cell memory formation has been much expanded (47), and it is now known that differences in the activation microenvironment and TCR signal strength are crucial for the fate decision process (15, 24). The cytokine milieu influences genome accessibility and the pattern of gene expression. These events are believed to take place through differential activity of chromatin remodeling complexes, transcription factor balance and modulation of proteasome activity (12, 13, 15, 48–50). That, together with the duration of TCR signaling may determine the depth and reversibility of chromatin changes (43). *In vitro*, exposure of differentiating CD8 T cells to mild-avidity TCR engagement generates bias towards memory-like CD8 T cell differentiation, whereas intense TCR contributes to the differentiation of effector-prone cells (9–11). However, among the variety of memory cell subsets described *in vivo*, only cells presenting the “effector memory-like” phenotype have so far been generated *in vitro*. In a closer attempt, memory cells capable of surviving for 70 days post activation have been described, and despite being able to respond in recall responses, their protective ability and potential to improve the recipient’s health was not addressed (18). We therefore evaluated two classical properties of memory cells in our model: longevity, stemness and long-term protection. We generated stable memory cells, which were capable of maintaining their numbers and phenotype for over 30 days in culture (**Figure 5**), identifying a memory cell subset (CD44^hi^CD62L^lo^) that retain stem properties and gave rise to the more complex memory cells population when cultured (**Figure 11**). Moreover, adoptive transfer of these cells decreased the bacterial load and promoted the survival of 28% of mice challenged 20 days after T cell transfer (**Figures 12D–F**). In addition, the lack of bacterial control in the group that received effector cells suggested that *in vitro*-generated effector OT-I cells resembled terminal effector cells, and we were not able to give rise to a memory population upon *in vivo* transfer (**Figures 12D–F**). Even though the *in vivo* persistence and functional *in vitro* response of memory-like *in vitro*-generated memory cells has been previously reported (18), here we show for the first time the actual protection and half-life increase of infected mice following this treatment (**Figures 12E, F**). Transfer of CD44^-^ naïve CD8 T cells assures that bacterial clearance is not mediated by CD44^hi^CD122^+^ virtual memory lymphocytes (51). Moreover, we were able to reduce the growth rate of established B16-OVA melanoma through the adoptive transfer of *in vitro*-generated memory OT-I cells, demonstrating the potential use of this protocol for anti-cancer immunotherapy. The lack of accessory cells provides advantages in purity and for applications where scaling up the protocol may be required.

Effector and memory lymphocytes have distinct gene signatures that dictate the differentiation destiny of CD8 T cells (13, 52–54). Most of the work characterizing effector and memory lineage precursors thus far has been done on cells isolated from *in vivo* studies of virus-infected animals, often utilizing T lymphocytes from TCR transgenic animals (13, 38, 40, 55). Recent work using TCR transgenic *in vitro*-labeled and *in vivo* transferred CD8 T cells has suggested that both phenotypes, effector and memory, are induced by differing expression signatures that have already been detected one division after stimulation and converge to a similar pattern of gene expression upon 4 and 7 days post-activation, before diverging again (13). Moreover, it has been suggested that memory cells undergo an effector-associated gene expression profile prior to committing to the memory phenotype and may arise from dedifferentiation gene expression reprograming (45). Despite the relevance of this model, the lack of markers that consistently distinguish early precursors of these two lineages limits mapping of cells early in the process of differentiation. Interpretation of these data is difficult and relies on the application of big data analysis filters to try to separate each population, intrinsically excluding information that does not fit a detectable pattern. As noted by the authors, the large number of transgenic cells transferred and method of labeling for isolation of cells that have undergone only one division may impinge selective pressure on the investigated cells (13). By improving a differentiation protocol exclusively *in vitro* that does not require antigen presenting cells, we have an ideal system for molecular analysis of early memory CD8 T cell precursors, which can then be compared to early effector cell precursors. Memory CD8 T cells generated in our *in vitro* protocol showed high expression of *Tcf7*, *Id3*, *Bcl6* and *Eomes*, genes that are classically associated with the memory phenotype, as well as low levels of the effector-related genes *Prdm1*, *Hk2*, *Zeb2* and *Ezh2* (**Figures 4**, **9**). The maintenance of *Tbx21* expression at levels close to those of naïve cells is in accordance with previously published data (12). Furthermore, the gene expression profile of a single cell after LCMV infection demonstrated that the *Tbx21* mRNA levels were similar among effector, central and effector memory cells, but slightly higher in the memory population (13), strengthening the *in vitro* model developed in this work, and emphasizing the importance of *Tbx21* vs. *Eomes* expression levels for cell fate decisions.

Moreover, the analysis performed along the *in vitro* memory differentiation protocol showed that CD44^+^CD25^hi^CD127^-^CD8^+^ cells were capable of producing high levels of granzyme B and IFN-γ and showed high expression of genes related to the effector profile between the 3^rd^ and 5^th^ day (**Figure 8C** and **Supplementary Figure 3B**). These data were similar to the kinetic analysis of effector cells (**Figures 8**, **9**), reinforcing the hypothesis that memory cell precursors acquire effector characteristics prior to consolidation of their mature phenotype and corroborating previously reported data suggesting a role for precursor memory effector cells (MPECs) in this process (12, 45, 56, 57).

Consistent with the phenotypic and molecular similarities between effector and memory CD8^+^ T cell precursors, we demonstrated that these cells were plastic, and if exposed to the right cytokine cocktail, their differentiation paths could change (**Figure 10**). These characteristics suggests that CD8 T cells maintain a high degree of adaptive capacity, such that changes in the host’s immune condition or antigen context may skew the differentiation of effector or memory cells even after the initial activation.

Finally, analyzing the mature memory cells generated herein, we identified the CD44^hi^CD62L^lo^ population, which remain ill-defined but, as we showed, capable of a long lifetime and giving rise to the more classical CD44^hi^CD62L^hi^ population (**Figure 11**). This intrapopulational diversity is identified through molecular and functional parameters such as CD62L, a protein that is differentially expressed between central memory and effector memory cells (1–3). In our protocol, we further characterized these cells as long-lived and presenting the CD44^+^CD62L^lo^CD25^lo^CD127^+^ profile (**Figures 5**–**7**), characteristic of effector memory cells (3). These data suggest that the *in vitro* protocol generated both central memory and effector memory cells, which equally persisted throughout the *in vitro* differentiation process. Whether the mechanisms underlying this plasticity are similar to those previously described for effector-to-memory differentiation remains to be shown (16, 45).

Nevertheless, the memory CD8 T cells generated *in vitro* using our protocol were able to confer protection to naïve mice against established tumors, delaying the progression of subcutaneously transferred, overly aggressive B16 melanoma cells (**Figures 12G, H**). Our data suggest that memory cells are even more efficient than *in vitro*-generated effector cells in this task (**Figure 12**), corroborating previous reports (58–61). The strong aspect of our protocol is the efficient expansion of this functional population that we have shown to be different from previously *in vitro*-differentiated cells, more similar to those generated *in vivo*. The 50-time expansion of CD8 T memory cells during *in vitro* culture allows for the collection of limited numbers of naïve cells from patients (**Figure 1B**, starting from 1 x 10^6^ naïve CD8 T cells, the protocol yields 16 ml of culture containing 3 x 10^6^ memory cells per ml after 10 days of culture). The stable expansion in the absence of heterogeneous accessory cells facilitates the translation of this protocol to the clinics.

In conclusion, we generated an *in vitro* differentiation protocol that gives rise to functional memory CD8 T cells. With this tool at hand, we corroborated and expanded data suggesting that memory cells go through an effector-like state prior to achieving a mature memory phenotype. We suggest, that in addition to the two transcriptional programs previously described that characterize effector and memory CD8 T cells, we may have identified a third program common to recently and plastic activated CD8 T cells. Moreover, we have shown that T_EM_ cells are capable of generating T_CM_ cells *in vitro*, suggesting that either T_EM_ are naturally kept in a less differentiated state, or dedifferentiation steps can be elicited upon homeostatic, cytokine-induced survival and proliferation. Importantly, although it remains to be shown their pattern of tissue colonization, *in vivo* functional experiments show that the memory cells generated herein are able to slow pathology. When adoptively transferred prior to exposure these cells confer resistance to lethal bacterial infection, whereas when transferred after tumor onset, to tumor-bearing mice, these cells confer resistance to tumor growth. Due to the ease of scaling up the cultures described herein, with the generation of up to 50 times the initial cell usage, and their demonstrated memory phenotype, self-renewal properties, capacity of generating effector-memory cells and *in vivo* protective therapeutic effects, these protocols may be especially useful in the generation of cells for cellular immunotherapy and large-scale molecular analysis.

## Data Availability Statement

The original contributions presented in the study are publicly available. This data can be found here: https://www.ncbi.nlm.nih.gov/bioproject/?term=PRJNA813210.

## Ethics Statement

The animal study was reviewed and approved by Ethics Committee of Animal Experimentation from INCA (CEUA process nos. 004/13 and 008/13).

## Author Contributions

VN-M performed most experiments, analyzed the data and wrote the manuscript. CC performed experiments and gene expression analysis. GM performed RNA-seq analysis. BF performed tumor experiments. LM performed tumor experiments. EC performed tumor experiments. ANAG performed RNA-seq analysis. HN proposed and interpreted RNA-seq analysis. RP planned and performed experiments of LCMV infection, RNA-seq, interpreted results and reviewed the manuscript. MW planned and performed experiments, analyzed the data, interpreted results and wrote the manuscript. JV planned experiments, analyzed the data and wrote the manuscript.

## Conflict of Interest

The authors declare that the research was conducted in the absence of any commercial or financial relationships that could be construed as a potential conflict of interest.

## Publisher’s Note

All claims expressed in this article are solely those of the authors and do not necessarily represent those of their affiliated organizations, or those of the publisher, the editors and the reviewers. Any product that may be evaluated in this article, or claim that may be made by its manufacturer, is not guaranteed or endorsed by the publisher.

## Funding

This work was supported by grants to JV from CNPq (408127/2016-3 and 307042/2017-0) and FAPERJ (203.007/2016), and grants to MW from CNPq (479022/2013-5) and FAPERJ (111.274/2014, 203.277/2016 and 010.002250/2016). VN-M was supported by fellowships from INCA and CC and BF were supported by fellowships from CNPq.
