# Supplementary material for: Differentiation of Memory CD8 T Cells Unravel Gene Expression Pattern Common to Effector and Memory Precursors
Source: Front Immunol. 2022 May 23;13:840203. doi: 10.3389/fimmu.2022.840203 (PMC9168330; doi:10.3389/fimmu.2022.840203)
Supplement: Supplementary file 1 [file DataSheet_1.pdf]

Supplementary Material

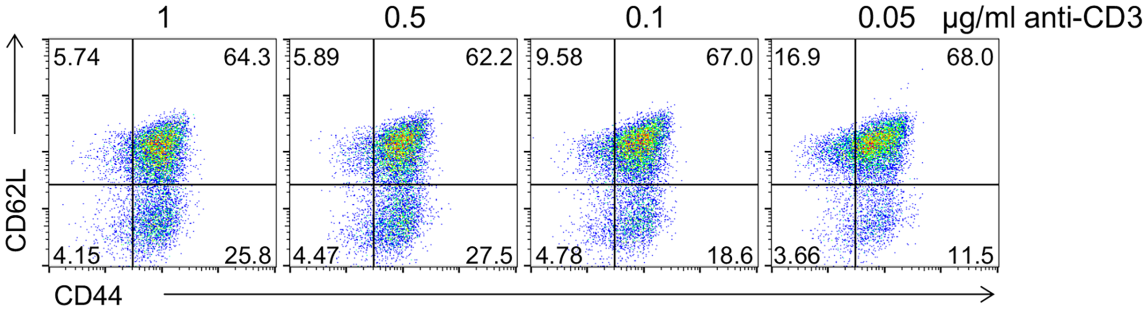

SUPPLEMENTARY FIGURE 1. Lower *In Vitro* TCR Stimulus Favors the Differentiation of CD62L^{hi} CD8 T Cells. Total CD8 T cells were activated *in vitro* with different concentrations of anti-CD3 (1, 0.5, 0.1 and 0.05 µg/ml) plus 1 µg/ml of anti-CD28 in the presence of IL-7 and IL-15 (both at 10 ng/ml). Cells were analyzed by FACS using CD44 and CD62L fluorescently labeled monoclonal antibodies after 8 days of culture. The results are representative of three independent experiments.

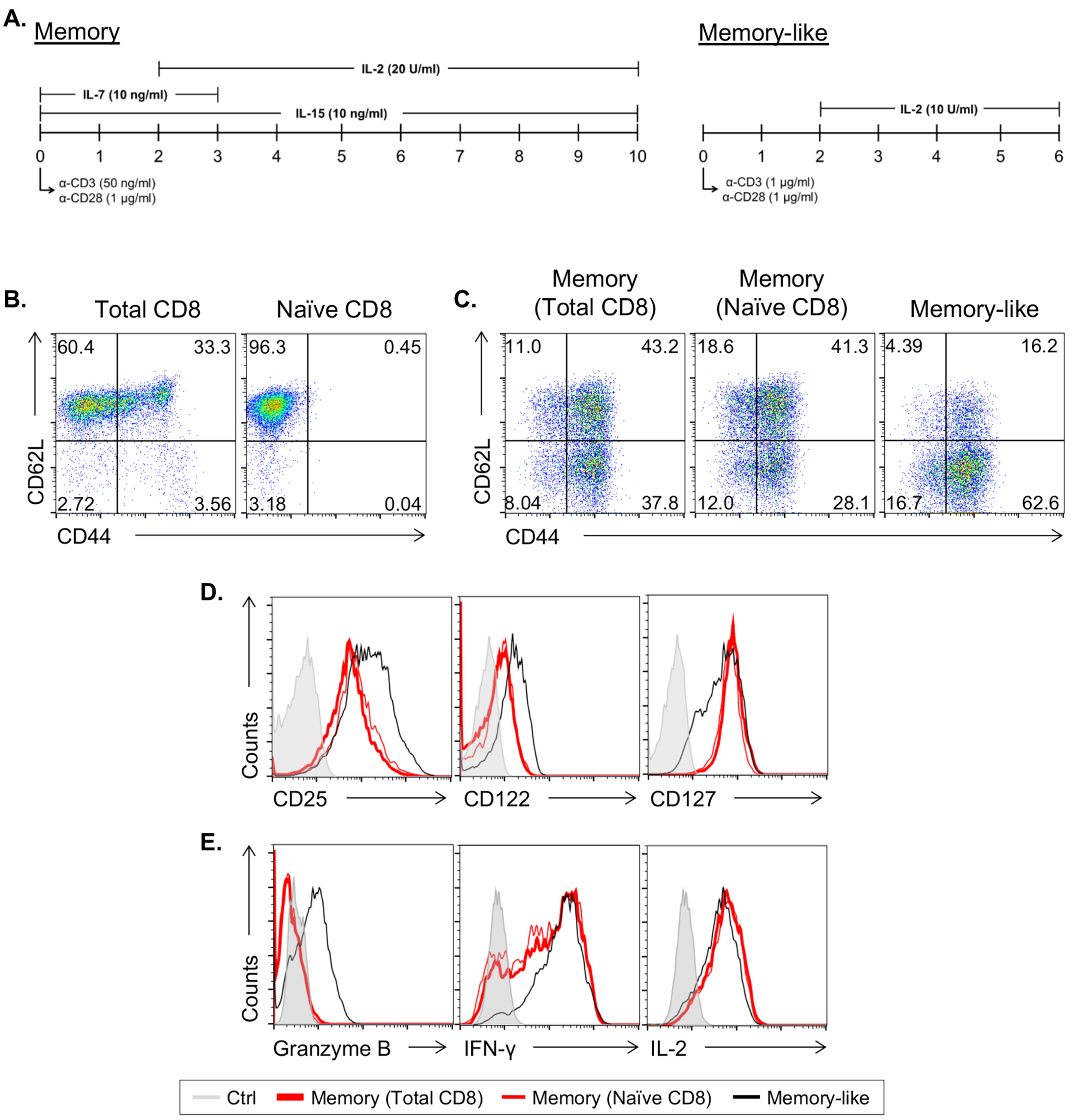

SUPPLEMENTARY FIGURE 2. Culture of Isolated CD8 T Cells in the Presence of IL-7 and IL-15, in Addition to IL-2, is More Efficient in Generating Functional Central Memory CD8 T cells *In Vitro*. **(A)** Schemes of *in vitro* memory cell culture protocols developed by our group (left) and previously published (right). **(B)** CD8 T cells were isolated as a whole population (total CD8) or sorted for $CD8^{+}CD44^{-}$ (naïve CD8) and cultured under both protocols depicted in A. **(C)** Analysis of CD44 and CD62L surface levels by total CD8 T cells and naïve CD8 T cells cultured for 10 days in the presence IL-2, IL-7 and IL-15 (as in A, left), or naïve CD8 T cells cultured for 6 days in the presence of only IL-2 (as in A, right). **(D)** Analysis of CD25, CD122 and CD127 levels by memory cells after 10 days of differentiation in the presence IL-2, IL-7 and IL-15. **(E)** Differentiated lymphocytes were stimulated with PMA and ionomycin for 6 h and ICC stained for IFN-γ, granzyme B and IL-2. The results represent one experiment. Ctrl: unlabeled (gray); memory from total CD8 (thick red); memory from naïve CD8 (thin red).

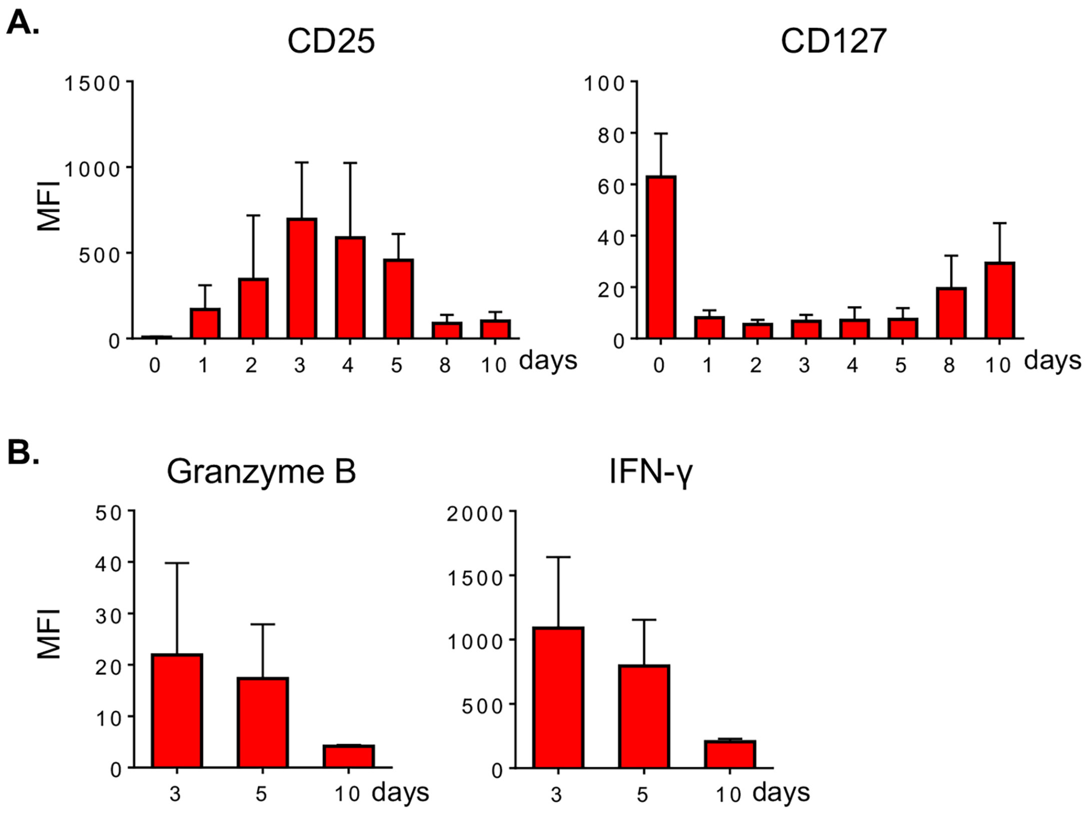

SUPPLEMENTARY FIGURE 3. Characterization of *In Vitro*-Generated Memory Cells during the Differentiation Process. Analysis of activated lymphocytes during the memory differentiation process from Fig. 5. **(A)** MFI of CD25 and CD127. **(B)** Lymphocytes activated *in vitro* were challenged with PMA and ionomycin for 6 h and ICC stained for IFN-γ and granzyme B at day 3, 5 and 10 of memory culture. All data are shown as the mean ± SD. The results are representative of three independent experiments.
